# Carbon monoxide poisoning in the 21st century

**DOI:** 10.1186/cc13846

**Published:** 2014-04-28

**Authors:** Angela L Chiew, Nicholas A Buckley

**Affiliations:** 1 Clinical and Experimental Toxicology Unit, Department of Emergency Medicine, Prince of Wales Hospital, Barker Street, Randwick, NSW 2031, Australia; 2 Pharmacology, Sydney Medical School, University of Sydney, Sydney, NSW 2006, Australia; 3 NSW Poisons Information Centre, The Children’s Hospital, Westmead, Sydney, NSW 2145, Australia

## Abstract

The world has experienced some very large shifts in the epidemiology of carbon monoxide poisoning, but it remains one of the most important toxicological global causes of morbidity and mortality. The diagnosis can be quickly confirmed with blood gases (pulse oximeters lack both sensitivity and specificity). Several strong predictors for serious neurological sequelae (prolonged loss of consciousness and elevated S100B) and reduced life expectancy (elevated troponin) are now reasonably well established. Despite this clearly defined high-risk group and extensive research into the pathophysiology, there has been little translation into better treatment. Much of the pathophysiological research has focused on hyperbaric oxygen. Yet it is apparent that clinical trials show little evidence for benefit from hyperbaric oxygen, and the most recent even raises the possibility of harm for repeated courses. More logical and promising potential antidotes have been under-researched, although recently both animal and small human studies suggest that erythropoietin may reduce S100B and prevent neurological sequelae. Major breakthroughs are likely to require further research on this and other treatments that may inhibit post-hypoxic inflammatory responses and apoptosis.

## Review

Carbon monoxide (CO) results from the incomplete combustion of carbon-containing substances and is a colorless, odorless, and tasteless gas [1]. Exposure is most commonly from car exhaust (unleaded petrol cars produce about one tenth the amount of CO of older cars), faulty heaters, fires, and industrial accidents.

It is clear that the epidemiology is driven by the source of CO and the diagnostic definitions applied. Notably, in the US, there appears to be quite a different pattern, with extremely large numbers of patients diagnosed but a relatively low death rate; for example, it was estimated that there were 1,000 to 2,000 accidental deaths due to CO exposure each year, resulting from an estimated 50,000 annual exposures [2]. The US case fatality of less than 5% for a poisoning that is highly lethal in most other countries may reflect several factors. Firstly, poisoning is largely from domestic heating (compare with fire or suicide) and is diagnosed (and treatment given) sometimes in the absence of any significant elevation of carboxyhemoglobin (COHb). For example, poison center calls in the National Poison Data System (2000 to 2009) found that 45.1% of the 68,316 unintentional, non-fire-related, CO exposures were managed at the site of exposure (that is, without any confirmation) [3]. Even of those attending a hospital and referred for hyperbaric oxygen (HBO), 10% had an initial COHb within the normal range for heavy smokers (<10%) and less than 1% had a recorded COHb over 50% [4].

This experience contrasts with most other countries where fires and suicidal poisoning (car exhaust or charcoal) are the leading causes of diagnosed CO poisoning. In Australia, for example, accidental poisoning accounts for less than 10% of calls about CO poisoning and the deaths often outnumber admissions [5]. It is therefore much easier to compare countries and examine trends over time by examining trends in CO poisoning deaths. Several Asian countries are facing rapidly evolving epidemics of CO poisoning from suicide by charcoal burning [6]. This increase occurred in the late 1990s, after a much publicized case in the media of a 38-year-old Hong Kong woman who committed suicide in a sealed bedroom by burning barbecue charcoal. The media depicted this means of suicide as painless and non-violent. Subsequently, in Hong Kong and Taiwan, there has been an increasing incidence of charcoal burning suicides, which has led to an overall increase in the suicide rate of approximately 20% [6,7]. A strategy to reduce the risk of misuse of charcoal burners is urgently required. Previous experience with the removal of coal gas and catalytic convertors has demonstrated that means restriction can be extremely effective in reducing CO suicides [8-10]. A range of possible solutions may need to be tried given the practical difficulties of restricting suicidal use of widely available domestic cooking equipment. On a more positive note, many countries have seen a substantial reduction in lethal car exhaust poisoning with the rise of catalytic converters [8,11].

## Mechanisms of toxicity

The most clearly established mechanisms for CO toxicity relate to tissue hypoxia. CO binds to hemoglobin with an affinity 200 to 240 times that of oxygen [12]. As COHb cannot carry oxygen, this leads to reduced oxygen-carrying capacity. Furthermore, COHb increases the affinity of the remaining sites for oxygen, meaning that oxygen release by the remaining oxygenated hemoglobin is also impaired. Thus, CO leads to impaired oxygen delivery to tissues and eventually to marked tissue hypoxia when compensatory mechanisms to maintain oxygen delivery fail.

However, tissue hypoxia is not the only possible explanation for the toxic effects seen. CO causes harm by both oxidative stress that follows a period of hypoxia and cellular damage by inflammatory processes. CO binds to and inhibits mitochondrial cytochrome oxidase, thereby directly inhibiting aerobic metabolism (analogous to the effect of cyanide). In the brain, CO binds to cytochrome c oxidase, which results in impairment of ATP synthesis and increased production of reactive oxygen species [13,14]. Inflammatory changes in acute CO poisoning include intravascular neutrophil activation due to interactions with platelets. This leads to neutrophil degranulation and perivascular oxidative stress [15]. While in rat models, CO exposure has been shown to precipitate abnormalities in myelin basic protein due to reactions with lipid peroxidation products [16]. Furthermore, some damage may be caused by the marked oxidative stress, free radical production, inflammation, and apoptosis seen when oxygenation improves and CO concentrations fall after severe poisoning (analogous to reperfusion injury) [15].

Unfortunately, much of the evidence on these effects has not used positive controls (that is, hypoxic injury not due to CO), and it is unclear whether such processes differ in any meaningful way from similar effects seen with reperfusion injury [17]. It is clear that organs with the highest oxygen demand are the most susceptible to injury, and brain and cardiac effects dominate acute clinical features and also risk assessment for late or permanent effects. There is potential for both serious delayed neurotoxicity (including Parkinsonism and memory and concentration impairment) and cardio-toxicity (myocardial injury and reduced life-expectancy).

**Figure 1:**
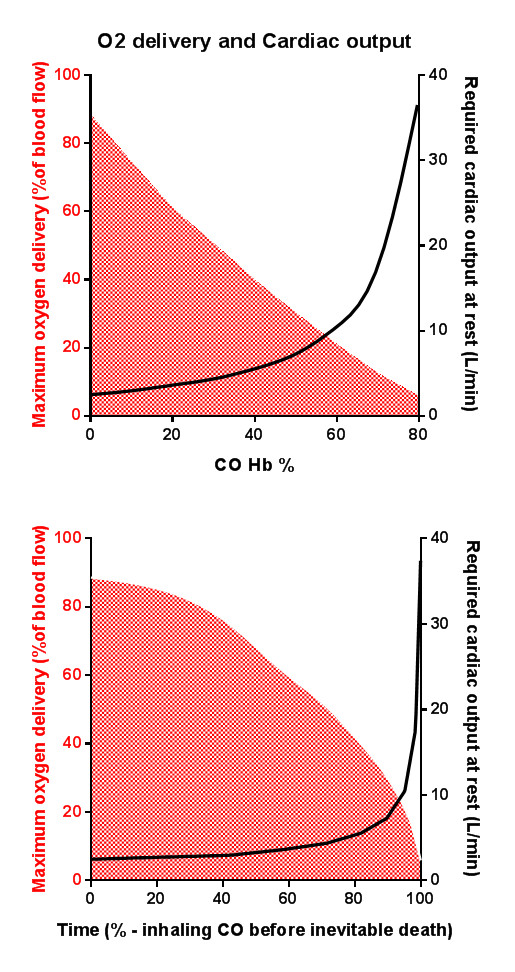
**Carboxyhemoglobin (COHb) versus oxygen delivery capacity of the blood and increased cardiac output required to deliver the same amount of oxygen. (a)** This is shown with a linear progression in COHb. **(b)** In the more likely scenario, there is continuous exposure to COHb and the rate of uptake of COHb is roughly proportional to cardiac output, resulting in a very rapid deterioration to life-threatening poisoning, after a relatively long mild poisoning stage. CO, carbon monoxide; O_2_, oxygen.

It is also apparent that many of the early non-specific clinical effects (for example, headache, nausea, and tachycardia) occurring at COHb concentrations of less than around 40% are likely to be signs of compensatory homeostatic responses rather than tissue hypoxemia. To maintain oxygen delivery to the brain, a large compensatory increase in cardiac output is required (as at altitude or with anemia). Unfortunately, any rise in cardiac output and respiratory rate also greatly accelerates CO uptake. At a critical COHb and time point, which likely varies considerably between individuals, the heart becomes unable to deliver a cardiac output great enough to compensate for the reduced oxygen-carrying capacity. At this stage, cardiac hypoxia will reduce cardiac output and exacerbate severe tissue hypoxia and death will rapidly occur unless there is intervention (Figure 1). The signs and investigations indicating that this critical tipping point has been passed (that is, more than transient loss of consciousness and elevated neuronal or cardiac injury biomarkers) have all been established as strong markers of short- and long-term prognosis [18-20]. The ability of the individual to compensate for the decreased oxygen-carrying capacity determines the level at which these more severe manifestations become apparent, and those with underlying anemia or respiratory, cardiac, or vascular disease may develop severe toxicity at lower concentrations.

## Long-term sequelae and prognostication

The long-term consequences in survivors can range from severe brain damage (which is fortunately uncommon) to a much more common syndrome of less severe but persistent problems. Neurological sequelae are often divided into persistent neurologic sequelae (PNS) and delayed neurologic sequelae (DNS) [1,21]. The incidence of neurological sequelae depends very much on the definition applied, the extent and timing of the assessment, and the population studied.

The concept of persistent neurological injury after a hypoxic brain injury is straightforward. DNS, in contrast, lacks a consistent definition, diagnostic criteria, or an established mechanism. However, the apparent development of the first neuropsychological symptoms or signs occurring days to weeks after CO poisoning clearly occurs. DNS varies very widely in studies from a few percent to two thirds of patients [22-25]. However, the ratio of DNS to PNS appears much lower when studies are prospective and careful monitoring is done from the beginning (that is, DNS may sometimes reflect delayed diagnosis rather than delayed appearance of symptoms).

Neurological sequelae are most commonly subjective and affect mood, short-term memory, attention, and concentration. The most common problems encountered are depressed mood (even in those accidentally exposed) and difficulty with higher intellectual functions (especially short-term memory and concentration). More severe problems include areas typically affected by ‘watershed’ infarcts (for example, basal ganglia and memory). In some cases, these are not noted initially but present later after initial recovery (typically within a week of the exposure). Neuro-psychological testing may be useful to provide objective measures of subtle deficits not found with routine bedside mental state examination and also to monitor the progress of these sequelae. Long-term follow-up is necessary in those at risk, as more subtle defects can develop or become apparent over a few weeks to months. However, the long-term prognosis is favorable in the majority of cases, and symptoms gradually resolve over the first few months [26], and the overwhelming majority of patients with CO poisoning return to full-time work [27].

Identification of patients who are at risk of neurological sequelae serves an important role in terms of counseling and indicating the extent of follow-up warranted. The best identified risk factors for long-term neurological effects are early and obvious neurological damage or a sustained loss of consciousness during the CO exposure. Most studies have found that significant neuropsychological sequelae are confined largely to those who have loss of consciousness at some stage [27-29]. However, if less stringent criteria are used for neurological sequelae (that is, slightly low test scores), other risk factors are thrown up by univariate analysis (for example, prolonged or repeated exposures and older age). These risk factors may also represent confounding, or reverse causality (for example, older age is linked to a higher risk of poor memory and executive function irrespective of CO poisoning; and impaired cognition prior to exposure is a risk for prolonged or repeated CO exposures) [28]. The recent promulgation of such criteria as age of more than 36 years (irrespective of the absence of other more established risk factors) to guide risk assessment [26,28] or to alter treatment has little to recommend it; it greatly inflates the numbers perceived to be at risk and goes against the much stronger evidence of a relatively benign long-term outcome for CO poisonings without the established risk factors.

However, other objective ways to identify patients at risk of sequelae are also required, as the history of loss of consciousness may be complicated in some settings. Neuron-specific enolase (NSE) is a glycolytic enzyme that is localized primarily to the neuronal cytoplasm in the central nervous system. S100B is a calcium-binding protein localized to astroglial cells [30]. They are both released after hypoxic damage as a result of neuronal and astroglial cell death [31]. These markers show considerable promise as intermediate outcome measures for brain injury in both animals [32] and humans [19].

Studies to date in acute CO poisoning confirm this promise. In one recent Taiwan study, 10 out of 71 patients developed DNS. These patients not only had longer loss of consciousness but also had 15-fold higher S100B levels. Further statistical analysis demonstrated that this was an independent predictor of the development of DNS after acute CO poisoning: serum S100B of more than 0.165 μg/L predicted DNS with a sensitivity of 90% and a specificity of 87% (odds ratio 121, 95% confidence interval 4 to 3,467) [19]. The timing of S100B measurement is critical in interpretation, and high initial levels were associated with coma and cardiac injury but these dropped fourfold within 6 hours [33]. This and other studies have reported generally lower levels and only a minor association with CO poisoning with loss of consciousness but without such sequelae [34]. Further larger and long-term studies including more people with severe poisoning are required to clarify the optimal timing and threshold and the extent to which a normal S100B can be regarded as reassuring with respect to long-term prognosis. In general, results for NSE have shown a less obvious relationship to severity than S100B [33,34], although one study found that it was better linked to level of consciousness and also had a longer apparent half-life [30]. No studies to date have examined the relationship between NSE elevation and long-term sequelae.

## Toxicity assessment

The severity of poisoning is a function of the duration of exposure, the ambient concentration of CO, and the underlying health status of the exposed individual. COHb concentrations are a rough guide to the severity of exposure. Venous COHb levels predict arterial levels with a high degree of accuracy, and the difference between the two is unlikely to exceed 1% to 2% COHb [35].

Though useful for diagnosis when detected, the first measured COHb is not a reliable way to measure severity or predict long-term outcome [28,36]. The COHb measurement is often delayed. Furthermore, backwards extrapolation based on estimated half-life performs poorly. This is likely because of undefined factors in the highly variable elimination half-life, which depends on not only inhaled oxygen but also cardio-respiratory function. For example, isocapnoic-induced hyperventilation can increase CO elimination to the same extent as HBO [37].

The potential use of non-invasive pulse CO oximeters to obtain rapid, continuous, and field measurements for triage and monitoring has much appeal. There is a Food and Drug Administration-approved device (Masimo Rad-57 signal extraction pulse CO oximeter), but the pre-registration studies tested the accuracy of this machine using only healthy volunteers and COHb levels up to 15% [38]. Later clinical studies suggest that the machine should not be used, unless blood gases are not available. For example, in 120 patients presenting to an emergency department with simultaneous blood gases and pulse CO oximetry, the limits of agreement of the differences in measurement were −11.6% and 14.4% COHB. This greatly exceeds the ± 5% COHB defined as a clinically acceptable difference. Furthermore, a third of patients had a difference between their results of greater than ± 5% COHb, and the RAD-57 device detected COHb of only more than 15% with a sensitivity of 48%. Such results mean that the device clearly cannot be used to replace standard laboratory measurements or even triage those requiring formal measurements [39]. The role of this device may be restricted to situations in which detection of chronic low level exposures leading to COHb of less than 15% might serve an occupational health role.

The standard method to assess severity of exposure is to focus on neurological and cardiac symptoms indicating tissue hypoxia, such as loss of consciousness and chest pain. Objective evidence of ischemic damage may manifest with neurological signs, but detection of cardiac ischemia requires investigations looking for electrocardiography (ECG) changes and cardiac enzyme elevation (that is, troponin and CK-MB). Myocardial injury is very common, particularly in those with loss of consciousness or underlying vascular disease or both [40]. A prospective study of transthoracic echocardiography on 40 patients with CO exposure found that 50% had left ventricular systolic dysfunction. On repeat testing in 3 days, the majority (80%) had completely recovered [41]. These changes are not usually simply uncovering pre-existing coronary artery disease. In one small study, all coronary angiograms in 20 patients with elevated cardiac markers following CO poisoning showed normal coronary arteries [42].

Directly attributable long-term cardiac consequences (for example, ischemic cardiomyopathy) are unusual; however, a much higher mortality rate has been reported in those with a troponin rise after CO poisoning [20]. It is currently unclear whether this represents long-term cardiac sequelae or whether these markers occurred in those with underlying cardiovascular risk factors.

## Management

The widely endorsed aspects of management include removing the patient from the source of exposure and administering oxygen. Management should include the following:

•100% oxygen by non-re-breather mask (or ventilator) as soon as possible. Four to six hours of 100% normobaric oxygen will remove over 90% of the CO.

•Assessment of severity and potential for coexistent poisonings as above, including 12 lead ECG and electrolytes, full blood count, and COHb.

•Supportive care: ensure that the airway is maintained for those with impaired consciousness, with intubation if necessary; ensure that the patient is quiet and resting as unnecessary muscle activity increases oxygen demand.

•Obtain serial ECG and cardiac enzymes in patients with a history of sustained loss of consciousness, cardiovascular disease, chest pain, or ECG changes.

The main therapeutic goal is to prevent acute and chronic neuropsychiatric consequences. Oxygen is the most important treatment and is always indicated for at least 6 hours. Oxygen toxicity is unlikely with less than 24 hours of treatment. Currently, the evidence for any treatment beyond 100% oxygen is very weak [43].

## Hyperbaric oxygen

**Figure 2:**
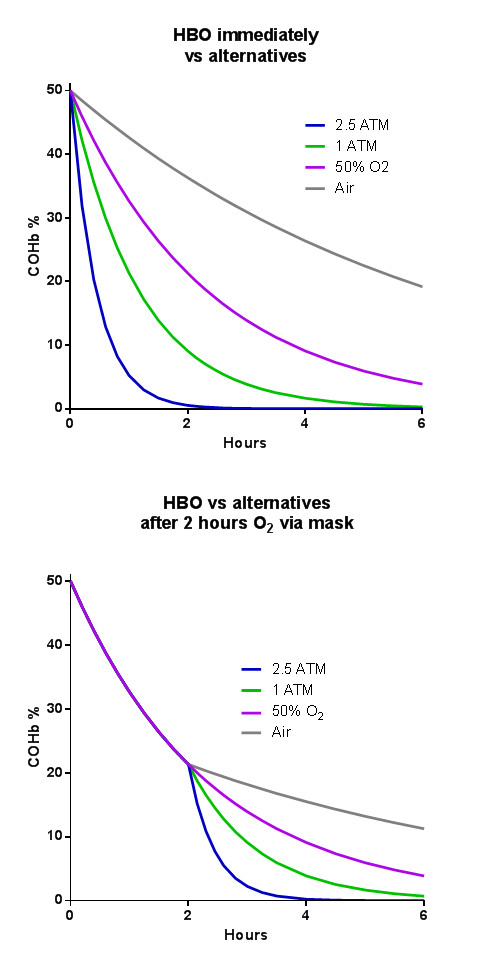
**Elimination of carboxyhemoglobin with various oxygen therapies. (a)** Theoretical elimination of carboxyhemoglobin (COHb) after removal from source and given hyperbaric oxygen (HBO) versus other oxygen therapies. **(b)** Elimination of COHb after removal from the source, receiving high-flow oxygen and following a typical delay of 2 hours to be given HBO versus other oxygen therapies. ATM, standard atmospheres; O_2_, oxygen.

HBO therapy is defined as the breathing of 100% oxygen by patients within hyperbaric chambers compressed to greater than 1.4 atm of absolute pressure. The half-life of CO in room air is around 4 to 5 hours. These half-life values decrease to approximately 40 to 80 minutes with administration of ‘100% oxygen’ and to 23 minutes when hyperbaric (2 atm) oxygen is used (Figure 2a). However, given that the half-life is only around 90 minutes with high-flow oxygen and that it typically takes at least 2 hours to arrange HBO treatment [4,44], the biological rationale that HBO is a more effective means of removing CO in practice is limited (Figure 2b).

It has been suggested that HBO may decrease the risk of neuropsychiatric sequelae due to other mechanisms not dependent on enhancing CO elimination. However, HBO might also feasibly increase oxidative stress during recovery and is substantially more expensive than normobaric oxygen. Furthermore HBO therapy can occasionally be complicated by barotrauma [21], seizures [45], pulmonary edema, and claustrophobia. It is contraindicated if there has been chest trauma or if the patient requires close monitoring or is non-cooperative.

The term HBO therapy covers a broad range of treatments; one survey of North American hyperbaric facilities found 18 different protocols. Among these protocols, the shortest period of compression lasts 46 minutes whereas the longest lasted 3 hours [46]. Some centers use multiple compressions over several days. All of these protocols are lacking evidence to support their choice over any other protocol or indeed that they improve outcomes in human poisoning.

**Figure 3:**
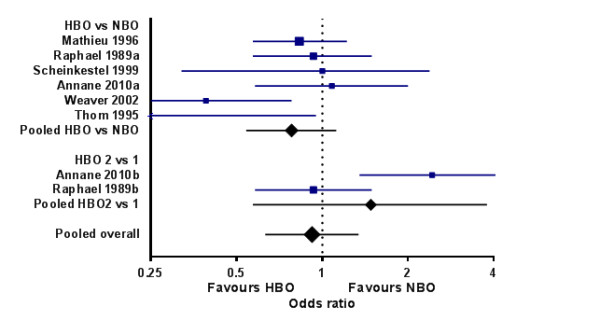
**Forest plot of treatment effects seen in randomized controlled trials of hyperbaric oxygen therapy.** HBO, hyperbaric oxygen; NBO, normobaric oxygen.

The stated goal of hyperbaric treatment is the prevention of long-term and permanent neurocognitive dysfunction; no study has yet demonstrated a reduction in mortality [47,48]. A 2011 systematic review (Cochrane collaboration) identified six randomized clinical trials that have compared HBO versus normobaric 100% oxygen [43]. Four of these studies reported negative and two positive outcomes at 4 to 6 weeks. The negative studies had mostly subjective outcome measures of neurological recovery and may feasibly have overlooked clinically important benefits. In contrast, it was evident that there was a high risk of bias from the approach to analysis applied in the positive studies. The largest positive trial was prematurely stopped ‘for benefit’ but despite this would have been a ‘negative’ study but for the numerous assumptions and protocol variations (including a change in the primary outcome) that all favored showing benefit from HBO [22]. Furthermore, a recent study comparing one versus two HBO sessions in comatose patients found more neurological sequelae in the group with repeated HBO [27] (Figure 3).

The role of HBO in the acute management of patients with very severe CO poisoning, such as coma, seizures, severe metabolic acidosis, or cardiac dysfunction, is particularly unclear as controlled clinical trials have frequently excluded such patients. If rapidly available, HBO may be the most effective mechanism for treating hypoxia in such patients; however, it may also be difficult to use unless a multi-person chamber is available so that medical/nursing care can continue uninterrupted.

Even among those advocating HBO, controversy exists about the indications for use of HBO in CO poisoning. Recommendations for the use of HBO in CO poisoning vary greatly between guidelines. The American College of Emergency Physicians clinical guidelines committee state: ‘HBO is a therapeutic option for CO-poisoned patients; however, its use cannot be mandated. No clinical variables, including COHb levels, identify a subgroup of CO-poisoned patients for whom HBO is most likely to provide benefit or cause harm’ [49]. Similarly, the National Institute for Health Care Excellence: Clinical Knowledge Summaries advise treatment with 100% oxygen, preferably via a face mask with reservoir. They do not currently recommend treatment with HBO, as ‘there is insufficient evidence’ that HBO ‘improves long-term outcomes of people with severe carbon monoxide poisoning, compared with standard oxygen treatment’ [50].

In contrast, the Undersea and Hyperbaric Medical Society has much broader recommendations and suggests HBO therapy for patients with serious CO poisoning, as manifested by transient or prolonged unconsciousness, abnormal neurologic signs, cardiovascular dysfunction, or severe acidosis, or for patients who are 36 years of age or older and were exposed for 24 hours or more (including intermittent exposures) or who have a COHb level of 25% or more [26]. Four authors who are prominent in this society have published similar recommendations [47].

In conclusion, HBO has a plausible but not compelling rationale, is of unknown effectiveness, and has some risks including that it might feasibly worsen neuropsychological sequelae. The risk/benefit will remain unclear until a large multi-center double-blind randomized controlled trial examining long-term clinical outcomes is performed; however, no such trials are currently registered as ongoing.

## Pharmacological treatments

A diverse group of pharmacological treatments has been investigated as treatment options in CO poisoning, with the aim of decreasing the rate of neurological sequelae. We have highlighted those agents that have already been proven safe for use in humans for other indications.

Erythropoietin (EPO) is a cytokine that originally was identified for its role in erythropoiesis and more recently was shown to be produced in the central nervous system. EPO offers neuronal protection when administered systemically to animals with global and focal cerebral ischemia. EPO administration led to substantial dose-dependent reduction in S100B following CO poisoning in an animal model [51]. A randomized prospective study of 103 patients with CO poisoning compared subcutaneous EPO for a week with placebo. S-100β levels decreased more rapidly in patients in the EPO group, and stroke scores were also better. At 30 days, fewer patients in the EPO group had delayed neurologic sequelae (12% versus 30%, *P* = 0.021) [52]. However, enthusiasm should be tempered by the experience with EPO in stroke, in which favorable small studies were followed by a large negative study that showed a higher death rate in the EPO arm [53].

Another novel treatment that has been repeatedly studied in animals for acute CO poisoning is hydrogen-rich saline. Hydrogen-rich saline is an anti-oxidant that at this stage is currently used in Japan for human metabolic disorders. It is non-toxic, convenient, and safe to use. It has been shown in rat studies to decrease neuronal necrosis and apoptosis, and improve neurobehavioral function, following CO poisoning. Its suggested mechanisms of action include reducing reactive oxygen species levels and upregulating endogenous anti-oxidative enzymes [54-56].

A diverse group of other substances has been tested in animal studies and has shown possible benefit, including granulocyte colony-stimulating factor, nimodipine, fructose diphosphate, hyperoxygenated solution and edaravone [57-60]. An even more diverse group of substances has been shown to be effective in hypoxic/reperfusion injury. The use of any of these agents cannot be recommended outside of clinical trials; however, there may be more rational treatments to test further in clinical trials to prevent neurological damage from reactive oxygen species than HBO.

## Conclusions

The mainstay of management of CO poisoning involves early 100% oxygen therapy. It is important to identify patients at high risk of neuropsychological sequelae, and the best established predictor is prolonged loss of consciousness. However, new biomarkers such as S100B show considerable potential to improve this prediction. Better validated objective predictive tools would greatly assist in the assessment of new and old treatments. The benefits, risks, and indications for HBO remain unclear despite six randomized controlled trials. Pharmacological treatments that reduce reperfusion injury and apoptosis, such as EPO, show considerable potential. These have a stronger biological rationale and would be more widely available, rapidly administered, and less expensive. Large multi-center trials with objective and functional long-term outcomes are needed for both old and new treatments before they can be recommended.

## Abbreviations

CO: Carbon monoxide; COHb: Carboxyhemoglobin; DNS: Delayed neurologic sequelae; ECG: Electrocardiography; EPO: Erythropoietin; HBO: Hyperbaric oxygen; NSE: Neuron-specific enolase; PNS: Persistent neurologic sequelae.

## Competing interests

The authors declare that they have no competing interests.

## Authors’ contributions

ALC and NAB performed the literature search, drafted the manuscript, and performed the revisions. NAB designed the figures. Both authors read and approved the final manuscript.
